# Supplementary figures and images for: Long-Term Clinical Outcomes of Left Atrial Appendage Closure in Patients with Left Atrial Appendage Thrombus
Source: J Clin Med. 2025 Oct 26;14(21):7589. doi: 10.3390/jcm14217589 (PMC12608996; doi:10.3390/jcm14217589)

## S1: Screening of Patients Referred for Left Atrial Appendage Closure

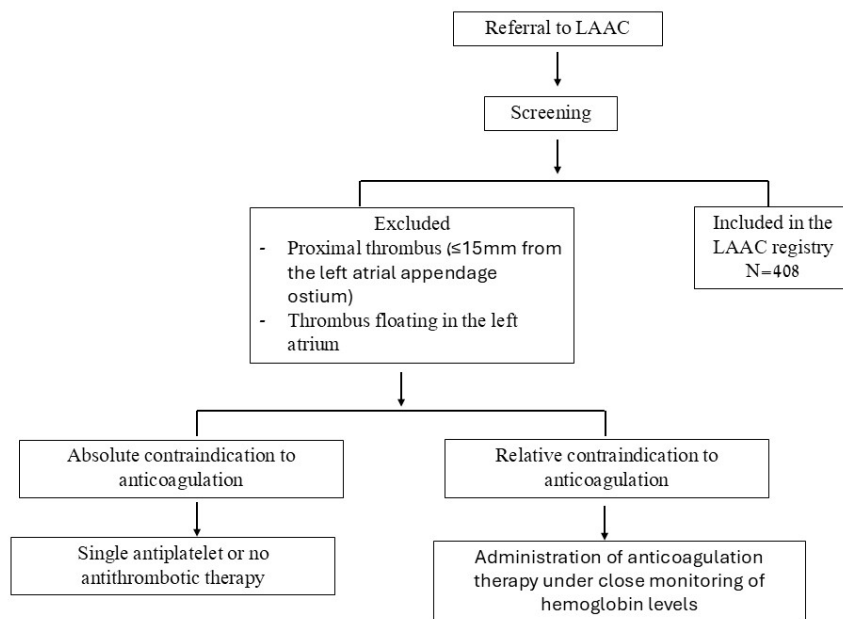

Supplement: Supplementary file 1 [file jcm-14-07589-s001.zip › jcm-3920643-supplementary.pdf]
